# Experiences of People Living with a Kidney Transplant: A Phenomenological Study

**DOI:** 10.3390/healthcare13222986

**Published:** 2025-11-20

**Authors:** Wichitra Kusoom, Narin Suwanboriboon, Sangnapa Siewthong, Sununta Krongyuth, Arunee Hengyotmark

**Affiliations:** 1 Faculty of Nursing, Bangkokthonburi University, 16, 10 Liabklong Taweewattana Rd., Bangkok 10170, Thailand; wichitra.kus@bkkthon.ac.th; 2 Department of Nursing, Bhumirajanagarindra Kidney Institute Hospital, Khet Ratchathewi, Bangkok 10400, Thailand; narinsusuk@gmail.com (N.S.); sangnapasiewthong33@gmail.com (S.S.); 3 Faculty of Nursing, Ubon Ratchathani University, 85 Sathonmark Road, Mueang Sri Kai, Warin Chamrap, Ubon Ratchathani 34190, Thailand; sununta.k@ubu.ac.th; 4 Office of the President, Navamindradhiraj University, 3 Khao Rd., Bangkok 10300, Thailand

**Keywords:** kidney transplantation recipients, physical and mental health, spirituality, phenomenological study, Thailand

## Abstract

**Background/Objective**: Kidney transplantation is the most successful treatment for patients with end-stage kidney disease. However, there are positive and negative impacts on physical and mental health. This study aimed to explore the life experiences of the effects of physical, psychological, and sociocultural aspects, and managing for resilience among people with KT. **Methods:** A descriptive phenomenological study was employed. Data were collected through in-depth interviews with 25 participants from the Bhumirajanagarindra Kidney Institute Hospital in Thailand. Thematic analysis was applied by using Braun and Clarke’s method. **Results:** The five themes, including (1) having new life and life satisfaction, (2) fear of kidney rejection and complications, (3) gratitude and spiritual practices, (4) concerns for the high cost of healthcare expenses, and (5) patience with self-management and resilience. **Conclusions**: We suggest that holistic, financial, and culturally congruent care should be implemented among people undergoing kidney transplantation to promote resilience and a longer life.

## 1. Introduction

Chronic kidney disease (CKD) is a health problem that has a significant impact on health, the economy, and society worldwide. Kidney transplantation (KT) is a successful treatment for improving survival rate and quality of life for patients. The Thai Organ Transplantation Society [1] reported in 2023 that there were 7555 KTs in 36 healthcare centers across the country, while 6187 patients with end-stage kidney disease (ESKDs) were on the waiting list. The KT recipients (KTRs) have increased freedom, independence, and a return to near normality, with improved quality of life, physical activity, and function compared with their pre-transplant lives. However, they described living with anxiety about the health of their transplant and fear that it may fail [2]. The 1, 3, 5, 7, and 10-year survival rates of KTRs were estimated to be 99.60, 97.30, 95.20, 74.60, and 87.90%, respectively [3], and they had a better health-related quality of life (HRQOL) than CKD patients [4,5]. A systematic review by Wang et al. [4] indicated that KTRs had similar or marginally higher HRQOL compared with patients receiving dialysis, regardless of whether they were on the waiting list. Meanwhile, the treatment process was complex, and post-transplant, they encountered various issues such as physical and psychological stress, changes in social support, and financial problems [6], which were likely a key driver of the limited HRQoL in this population [7]. A systematic review by De Pasquale et al. [8] suggested that people with KT were exposed to a high risk of psychiatric disorders with repercussions on the quality of life and the risk of rejection.

An immunosuppressive medication is essential throughout their lives to maintain the transplanted kidney function which can lead to infections, hypertension, and diabetes. The infections include viruses such as Cytomegalovirus (CMV) and BK virus (BKV). These viral infections are an important predisposing factor for allograft rejection after kidney transplantation [9]. De Boer et al. [10] examined the immunosuppressive drug-related side effect, which was most strongly negatively associated with both mental and physical HRQoL. A qualitative analysis of life and expectations post-kidney transplant by Tucker et al. [11] found that common themes included: improvements in quality of life, a return to normalcy, better health, and more energy. Concerns included: duration of graft survival, fears about one day returning to dialysis or needing to undergo another kidney transplant, comorbidities, future quality of life, and the cost and quality of their healthcare. Many recipients were grateful for their transplant, but some were anxious about the burdens that transplantation placed on their loved ones. Similarly, a qualitative study by Wube et al. [12] revealed both positive and negative psychological experiences among kidney transplant patients. Positive experiences included feelings of “rebirth,” thankfulness, freedom from dialysis, and enhanced self-efficacy. However, negative experiences included dependency, fear of the future, challenges with adherence to treatment, occasional regret, and fear that it may fail. Furthermore, there were high healthcare costs. The healthcare costs are correlated with worse outcomes and death in people with KT [13]. A study by Birkefeld et al. [14] found that 49.2% KT patients had a lifetime mental disorder, and only 14.5% KT patients with a current mental disorder reported being in treatment. Therefore, KTRs experienced challenges in physical, psychological, and economic aspects.

Coping strategies are necessary to help patients with KT return to their everyday lives or build resilience. The American Psychological Association (APA) defines “resilience” as the process and outcome of successfully adapting to difficult or challenging life experiences, especially through mental, emotional, and behavioral flexibility and adjustment to external and internal demands [15]. Similarly, a qualitative study by Chung [16] found that resilience attributes included physical health management, coping strategies for recovery, optimism, family support, social-environmental support, socioeconomic support, perception of stressful situations, and positive thinking during recovery. Therefore, qualitative research helps to understand phenomena within their context and uncover the complex social, cultural, and individual factors that influence health. Currently, there is a lack of qualitative studies exploring the experiences of survivors of KTRs in Thailand. The study employed a phenomenological approach, which provided a deep understanding of the phenomenon as experienced by several individuals [17]. This understanding is crucial in informing clinical decisions that aim to enhance patient care. It will be helpful to understand the difficulties experienced during therapy and to comprehend the physical, psychosocial needs, and management of long-term care as KTRs.

This study aimed to explore the phenomenon of resilience among kidney transplant recipients in relation to physical, psychological, and sociocultural aspects.

## 2. Materials and Methods

### 2.1. Design and Participant Recruitment

This qualitative study employed a descriptive phenomenological approach, which provides a deep understanding of a phenomenon as experienced by several individuals [17]. The phenomenological research is underpinned by the philosophies of Edmund Husserl and Martin Heidegger. They were two of the most prominent philosophers who spearheaded this movement, and subsequently, they developed their own distinct philosophical approaches and methods of inquiry as means to exploring and understanding the human experience [18]. From a philosophical perspective, phenomenology has gained importance in the nursing profession. Moreover, phenomenological nursing studies are legitimate and important for developing theoretically informed insights that promote the discipline of nursing [19]. Therefore, it could be highly effective in expanding knowledge about better care for KTRs and also providing information to both pre- and post-KTRs. The setting was the KT department at Bhumirajanagarindra Kidney Institute Hospital (BKI) in Bangkok, Thailand.

### 2.2. Sample

The study population consisted of adult patients with KT attending the KT department, Bhumirajangarindra Kidney Institute Hospital, Thailand. Purposive sampling techniques were used. Inclusion criteria included adults aged ≥18 years, who had received a kidney transplant and had been discharged from the hospital more than 6 months earlier.

They had normal vital signs, no symptoms of dyspnea, shortness of breath, fever, palpitations, and no cognitive impairment; they were also able to communicate in-depth during the interviews. Moreover, they were willing to provide rich information about their experiences of living with KT. Exclusion criteria included cough, fever, tiredness, shortness of breath, palpitation, cognitive impairment, eye, and hearing impairment.

After the strict screening, 30 were eligible. In a phenomenological study, data were saturated when gathering additional data no longer yielded new insights or sparked new understanding. That was an adequate sample [17]. Therefore, a sample size of 25 participants was used.

Instruments included semi-structured individual interviews, open-ended dialogue, field notes, reflective notes, and audio recording tape. Some of the questions were along the lines of “How is your health?”; “Could you please tell me about your health issues while living with kidney transplantation?”; “What are your experiences of living with KT?” How did you cope with your health issues? What is your quality of life? etc.

### 2.3. Protocol

The researcher conducting the interviews had no prior relationship with the participants. The first interviewer reviewed the research objective, the interview’s duration, and the procedure for answering questions for the key informants. Data were collected through observation and face-to-face in-depth interviews guided by the research questions. Moreover, reflexive notes were revised before each interview. Probing questions and closing instructions were incorporated [17]. All Observations were focused on key informants. Interviews were recorded with the key informant’s permission. Each interview took approximately 30 to 45 min and was conducted 1 to 3 times until the data collection was completed. The first interview was conducted on-site, and the second and third interviews were conducted by phone. However, three participants were interviewed twice, and another participant was interviewed three times. The study was conducted between April and July 2025.

### 2.4. Data Analysis

The thematic analysis method by Braun and Clarke was applied. Since thematic analysis is one of the most widely utilized methods for analyzing qualitative data, it offers a structured yet flexible framework for identifying, analyzing, and interpreting patterns of meaning within datasets [20]. Two researchers analyzed the transcripts. There were six steps [20,21] including (1) Familiarization with the data; active reading and rereading, transcription, initial note-taking, and reflective observations, (2) Generating initial codes; systematic line-by-line coding, iterative refinement, coded the same passages, building agreement through consensus (everyone coded the same materials independently, comparing how different members, refining the code definitions based on discussions, document decisions in the research journal—capture what the researchers decided and the reasoning behind each choice). The researchers compared and discussed until a consensus was achieved to generate subthemes and themes. (3) Searching for themes; clustering similar codes, identifying relationships, creating thematic maps, (4) Reviewing themes; revisiting coded data extracts, refining boundaries, creating and analyzing thematic maps, (5) Defining and naming themes; detailed thematic analysis, writing theme descriptions, selecting supporting extracts, and (6) Results and writing the report; synthesizing themes, integrating illustrative quotes, and writing interpretive commentary. The model served to answer the research questions and underscore the study’s contribution to knowledge. This step signifies the culmination of the analysis, encapsulating all the findings and insights derived from the data.

### 2.5. Rigor and Trustworthiness

Four criteria were considered: credibility, dependability, confirmability, and transfer ability, collectively contributing to trustworthiness [17]. Rapport and trust were established, and prolonged periods were spent conducting fieldwork. The interviewer carefully avoided using ideas to lead participants to express their experiences. Triangulation was conducted. The reflexive data, checked by the research team, were carefully collected to meet the confirmability criteria. The transcript was returned to the two participants for their review and comment. Dependability was enhanced through the debriefing of the data collection and analysis, which included the input of two external consultants in nursing and a Buddhist monk.

Regarding the research team, two researchers had a PhD and a Master of Nursing Science (M.N.S.), who had experience with qualitative and mixed-methods studies, while the other two researchers were Registered Nurses (RNs) working at the Kidney Institute Hospital, and trained in qualitative data collection and utilized multiple methods (e.g., shortened sentences, writing, and gestures). This study was conducted in accordance with the Consolidated Criteria for Reporting Qualitative Research (COREQ) guidelines.

### 2.6. Ethical Consideration

This project received ethical approval from the Research Ethics Committee for Human Subjects (BKI Ref. No. 1/2025) and complied with all ethical standards of research practice. All participants were informed and assured of confidentiality. And they knew and understood that they could withdraw from the study at any time without negative consequences. Participants who agreed to participate in the study had to provide written informed consent first. Interviews took place in a quiet, private room after participants provided verbal informed consent. During the interviews, family members or healthcare providers were with the participants. Participants were assured that all data would be kept confidential. We assigned a participant number to replace the participants’ names on the transcripts. All electronic data were stored in password-protected files on secure computers and accessed only by the researchers. The reflexive notes and audio recordings were stored separately in a locked drawer.

## 3. Results

1. The participants’ demographic characteristics consisted of 25 participants, who comprised 13 males and 12 females, aged between 28 and 77 (average 53.9 years). There are 5 participants with LDKT and 19 participants with DDKT. Most participants had complications (Table 1).
2. In a qualitative procedure, five themes emerged with 25 subthemes. The five themes included (1) having new life and life satisfaction, (2) fear of kidney rejection and complications, (3) gratitude and spiritual practices, (4) concerns for high cost to healthcare expenses (5) patience with self-management and resilience. (Table 2).

**Figure 1:**
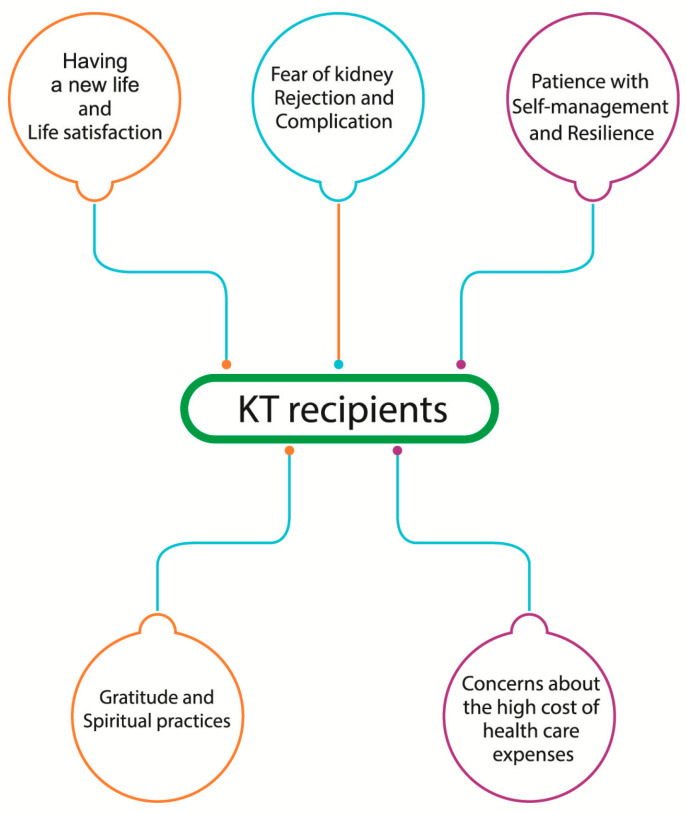
Model of resilience among kidney transplant recipients in relation to physical, psychological and sociocultural aspects, including (1) having a new life and life satisfaction, (2) fear of kidney rejection and complications, (3) gratitude and spiritual practices, (4) concerns about the high cost of healthcare expenses, and (5) patience with self-management and resilience.

From the themes to build a model of KTRs’ lived experiences of resilience (Figure 1).

1. Theme 1: Having a new life and life satisfaction:

Most participants were reported to be in significantly better health than those who underwent hemodialysis, and they returned to normalcy. They had higher energy and a new life or rebirth. They had the freedom to travel and move around independently. They feel that although they are unfortunate to have stage 5 ESRD, they are fortunate to have received KT with successful treatment results that are satisfactory and give them hope in life. Twenty participants’ life satisfaction is at its highest, four participants have a high level, and another participant has a moderate level of it. Five participants with live kidney transplantation (LKT) and two preemptive KTs reported normal health and extreme life satisfaction (score 10/10). Another participant experienced a kidney transplant failure 30 years after the transplantation (received a KT from another healthcare center). He later had a second kidney transplant more than 3 years ago (in 2022, received a KT from BKI). He is currently in good health and very satisfied with his life. (score 9/10). In addition, six KTRs aged 28 and 56 years have improved sexuality. And another case is a 33-year-old female (3 years and 10 months after a kidney transplant) who is planning to become pregnant.

*I am very satisfied with my life now. I am luckier than if I had won the first prize in the lottery. I feel better and healthier, and I am 100% better after 1 year and 8 months of surgery. I have more time to work and sleep well.*

(P…1)

*I had kidney failure from abnormalities in IgA and Hypertension, making it Stage 5 of kidney failure. My 26-year-old sister donated a kidney, so I had a quick kidney transplant without dialysis. I have now been on KT for 3 years and 8 months, and there is no need to use antihypertensive drugs; my IgA has returned to normal in every way, giving me hope in life. I have consulted with my husband and doctor about having children. The doctor said that I can have children, so I am adjusting my immunosuppressant medication and am planning to have children in the near future.*

(P…24)

2. Theme 2: Fear of kidney rejection and complications:

All participants worried about kidney rejection and complications. Nineteen participants experienced complications and comorbidities. They experienced worries about complications such as CMV, BKV, viral infection, kidney artery occlusion, stricture of the urethra, urinary tract infection, renal vein thrombosis, lymphocele, and drainage on the abdomen. Two participants had active antibody-mediated rejection (AMR) and a high level of creatinine. They underwent a kidney biopsy and plasmapheresis. Many participants had anxiety, fear, and stress due to infections, complications, and rehospitalizations that a nephrologist had treated. However, they received a kidney transplant more than one year ago, and feelings of relaxation, fears, and anxiety were reduced, and these problems were successfully treated with medical treatment. In this study, no participant experienced kidney rejection, and 22 participants returned to their baseline resilience. The other three participants experienced depression, suicidal ideation, and a high level of stress.

*I was worried about Kidney rejection and infection because of CMV and BK infections that were detected. I took three different kinds of medication per day. My anxiety has decreased a lot. I am now back on my feet again.*

(P…10)

*I was very stressed and uneasy about kidney rejection and abnormal blood test results. I had to do a kidney biopsy and six plasmapheresis sessions, but now that I’ve gotten through it, I feel much more at ease and think everything is satisfactory.*

(P…9)

3. Theme 3: Gratitude feeling and spiritual practices:

All participants expressed their gratitude and appreciation to the kidney donors. Participants had a profound sense of love for their beloved siblings or partners who had given them a live kidney. They felt grateful to the healthcare team and their family’s support. However, five participants experienced strange feelings such as smelling a lady’s perfume, delusions in the ICU, and a night dream. The participants believed that the kidney donor was taking care of their kidneys. In Thai culture, many participants decided to pray, practice mindfulness meditation, perform merit-making, and also donate money to the poor and charity organizations.

*To think positively in the face of this misfortune, there was also good fortune, such as having received a kidney transplant from a kind-hearted donor (without having known each other beforehand). There were many doctors taking care of me, including surgeons, immunosuppressant doctors, psychiatrists, and many nurses. There was a Line group network to provide advice on problems 24 h a day, which helped me overcome the difficulties. I felt warm and comfortable when I came to receive treatment at this hospital. I was grateful for the kindness that gave me new life once again.*

(P…8)

*I am very fortunate to have a good wife. In addition to giving me a kidney, she has always taken good care of me. She even went so far as to do things for me. I am mindful of her kindness and will perform good deeds and accumulate merit every month for my wife, the doctors, and the nurses.*

(P…13)

*I read Dhamma books every day without fail, make merit according to Buddhist beliefs, donate money to the poor and hospitals, give alms, pray, meditate, and practice Dhamma at home daily*.

(P…21)

4. Theme 4: Concerns about the high cost of healthcare expenses:

It was mentioned that the average cost of kidney transplantation ranges from 700,000 to 1,000,000 Thai Baht ($22,000 to $ 30,800). Additionally, immunosuppressants for anti-rejection were between 360,000 and 480,000 Thai Baht per year (between $11,000 and $14,700). The anti-rejection medication was essential because kidney recipients must take immunosuppressants for the rest of their lives with a functioning kidney graft. Some cases had complications that required rehospitalization, additional payments of 200,000–300,000 Baht. To cover such extra costs, most patients received government subsidies, support from their spouses, or assistance from their children or siblings, making the additional payments manageable. It was one of the causes of stress and insomnia, so they used anti-anxiety medication as prescribed by their doctor.

*The cost of surgery was high, but this hospital operates as a charitable healthcare Institution. The cost here was lower than in other places, but the service provided by the doctors and nurses was excellent. I had to be readmitted for another 7 days to do Plasma pheresis 4 times, costing more than 206,000 Baht. So, I felt very concerned.*

(P…14)

*I was worried that the funds would not be sufficient for the total amount of expenses.*

(P…24)

5. Theme 5: Patience with self-management and resilience:

All participants expressed their desire for a kidney transplant and their long-awaited outcome. The surgery was successful, and they accepted living with the transplant. The KTRs emphasized the importance of patience with self-management, returning to everyday life, and achieving life satisfaction. Most participants reported receiving advice on self-care and adherence to treatment. The participants complied with the advice of healthcare providers regarding fear of infection, kidney rejection, and death. Cultivating patience and strengthening the mind, along with positive thinking, helps one believe that everything will be fine. They described the various treatments, support from the healthcare team, and social support given to them, which resulted in their return to normal life one year ago. Therefore, twenty-two participants have returned to a normal life, healthier with improved quality of life and resilience. On the other hand, three participants had impaired resilience related to depression, suicidal ideations, a high level of emotional stress, and sleep disturbance. Medical treatment, psychotherapy, and supportive care were needed. Currently, the three participants are continuing to receive psychiatric treatments and supportive care for 1, 2, and 5 years, respectively.

*You must be patient and accept whatever may happen. Follow the advice of your doctor and nurses and help yourself as much as possible. Eat clean, cooked, and hot food. Avoid eating green leafy vegetables, seafood, shrimp, and shellfish. Avoid lifting objects that weigh more than 2 kg. Take your medicine on time and follow your doctor’s instructions strictly.*

(P…11)

*I had cystic kidney disease and had to have the cyst pierced through the abdomen to drain it. But now I’m better. The problem is gone. I’m not worried anymore. However, I must be diligent in observing myself and attending check-ups strictly.*

(P…12)

## 4. Discussion

The findings highlight the importance of participants having a new life and life satisfaction. Most participants reported an improvement in their health and a return to normalcy. A study by Antoun et al. [2] reported that individuals in these conditions experienced nearly the same level of freedom as those in normal conditions, an improved quality of life, and were able to perform physical activities more effectively. KTRs have a better health-related quality of life (HRQOL) compared to patients with CKD [4,5]. The positive experiences included feelings of “rebirth,” thankfulness, strengthened social bonds, freedom from dialysis, and enhanced self-efficacy [12]. A previous study found that the 1, 3, 5, 7, and 10-year survival rates of KTRs were estimated to be 99.60, 97.30, 95.20, 74.60, and 87.90%, respectively [3]. A systematic review by Wang et al. [4] indicated that KTRs had similar or marginally higher HRQOL compared with patients receiving dialysis, regardless of whether they were on the waiting list. However, the physical, mental health, and HRQOL were good in the first 1 or 2 years after receiving a KT.

Fear of kidney rejection and complications: All participants had fears and worries about kidney rejection and complications. The treatment process was complex, and post-transplant, they encountered various issues such as physical and psychological stress, changes in social support, and financial problems [6]. In this study, three participants had impaired resilience related to depression, suicidal ideations, a high level of emotional stress, and sleep disturbance. Currently, the three participants are continuing to receive psychiatric treatments and supportive care for 1, 2, and 5 years, respectively. A systematic review by De Pasquale et al. [8] suggested that people with KT were exposed to a high risk of psychiatric disorders with repercussions on the quality of life and the risk of rejection. Similarly, a study by Wube et al. [12] found negative experiences, which included dependency, fear of the future, challenges with treatment adherence, and occasional regret. Among KT recipients, older age, female sex, lower economic status, and more comorbidities were associated with increased depression risk. Many participants had infections, including cytomegalovirus (CMV) and Human Polyomavirus (BKV). These are crucial predisposing factors for allograft rejection after kidney transplant [9]. Another study indicated that older age, female sex, lower economic status, and more comorbidities were associated with increased depression risk. Incident depression after KT increased mortality, graft failure, and death-censored graft failure risks in KT recipients [14]. 

Having a gratitude feeling: Gratitude, thankful, or grateful is a feeling of appreciation (or similar positive response) by a recipient of another’s kindness. This kindness can be gifts, help, favors, or another form of generosity to another person [22]. In this study, all participants are Thai Buddhists who expressed their gratitude through spiritual and religious beliefs. Moreover, they participated in social activities which were related to merit-making by donating money and things to poor children, the hospital, the Foundation of Kidney, and monks to show gratitude to the donor. Gratitude in Buddhism is more than simply saying “thank you.” A deep appreciation arises from understanding the interconnectedness of all life. Buddhism teaches that every moment, every experience, and every person we encounter is part of a more extensive web of existence [23]. All major religions promote the concept of gratitude in some form. In Christianity, there is an emphasis on thanking God for His continued blessings and love, as echoed in the numerous psalms dedicated to praise and thanksgiving. Similarly, in Islam, the act of expressing gratitude, or ‘Shukr,’ is fundamental to a believer’s relationship with Allah, highlighting that acknowledgment of His gifts enhances spiritual growth. While Buddhism encourages practitioners to cultivate an attitude of gratitude, recognizing the interdependence of life and the contributions of others to one’s own well-being. Whether it is through prayers, rituals, or reflections, the essence of gratitude unites these diverse spiritual pathways [24]. A systematic review and meta-analysis by Diniz et al. [25] suggested that gratitude can be used as a therapeutic complement for treating anxiety and depression and can increase positive feelings and emotions in the general population. A study by Jiwattanasuk [26] found that gratitude towards the Buddha positively impacts behavior and psychological well-being. The appreciation of the Buddha was associated with their greater happiness and a heightened awareness of the value of life. Another study indicated that Buddhist practices involving gratitude, which were in turn related to mindfulness and meditative aspects, significantly improved emotional stability, strengthened personality, and reduced stress. Muslim gratitude practices were instrumental in reinforcing mental health, elevating happiness, and building resilience [27]. Furthermore, a qualitative review study of life and expectations post-kidney transplant by Tucker et al. [11] found that common themes included: improvements in quality of life, a return to normalcy, better health, and more energy. Concerns included: duration of graft survival, fears about one day returning to dialysis or needing to undergo another kidney transplant, comorbidities, future quality of life, and the cost and quality of their healthcare. Many recipients were grateful for their transplant but some were anxious about the burdens that transplantation placed on their loved ones. Therefore, gratitude practices in all religious contexts have been shown to improve psychological well-being, happiness, and resilience.

Concerns about the high cost of healthcare expenses: In Thailand, there are four main health insurance systems as follows: (1) Universal Health Coverage Scheme (UHC/Gold Card), (2) Social Security, (3) Civil Servant Medical Benefits or Government benefit, and (4) Private Health Insurance [28]. Each system has its own benefits. The UCS covered broader diseases except KTRs, but the people who have been working for the government or retirement as well as their couples and children are covered. This study consisted of participants with the Universal Health Coverage Scheme (UHC) and Civil Servant Medical Benefits. Participants who lacked government benefits had to cover their out-of-pocket expenses themselves. However, they were supported by their family, siblings, and the KT Foundation, BKI. A qualitative study by Lorenz et al. [29] confirmed that three main themes of treatment burden after kidney transplantation including: (1) patients must do to care for their health (e.g., attending medical appointments, taking medications), (2) stressors that exacerbate felt burden (e.g., financial concerns, health system obstacles) (3) impacts of burden (e.g., role/social activity limitations). Delayed graft function and previous sensitization were associated with increased costs post-transplantation [30]. Healthcare costs and complications were correlated with worse outcomes and death in people with KT [13].

In this study, self-management resulted in participants being resilient. Many participants had better health-related quality of life (HRQoL) than CKD patients. Self-management included medication adherence, dietary management, daily life management, and self-monitoring. It helped the KTRs reduce the long-term risk of complications [31]. A study found that self-management interventions were more effective than routine care in managing chronic diseases, significantly improving patients’ HQoL, self-efficacy, and reducing depressive symptoms [32]. The educational interventions were the most common self-management interventions and were provided 3 months to 1 year after receiving KT [33]. A study suggested that physical and mental health and HRQoL were good in the first 1 or 2 years after receiving a KT [4]. Therefore, the KTRs were physically and psychologically resilient. A qualitative study found that resilience attributes in patients with KT included physical health management, coping strategies for recovery, optimism, family support, social-environmental support, socioeconomic support, perception of stressful situations, and positive thinking during recovery [16]. Coping mechanisms, such as religious/spiritual approaches, social support, and participation in support groups, played pivotal roles in the recovery or resilience among patients with KT [34]. However, this study found that three participants had impaired resilience related psychological disorders (e.g., depression, suicidal ideation, high-level emotional distress, and sleep disturbance). Currently, the three participants are continuing to receive psychiatric treatments and supportive care for 1, 2, and 5 years, respectively. A study of psychological resilience among patients with chronic illness by Xu et al. [35] suggested that sleep quality and depression mediate the relationship between psychological resilience and quality of life in middle-aged and older adults hospitalized with chronic diseases. A study revealed that lower psychological resilience, lower education level, and higher cognitive distortions were factors associated with depression and anxiety in patients with ESRD [36]. A qualitative study [37] found that maintenance and promotion of self-management behaviors were (1) attentiveness to changes in one’s own body, (2) good partnership with medical care providers, (3) past painful experiences, (4) establishment of lifestyle habits, (5) autonomy to protect one’s own body, (6) support from family and others, (7) gratitude for kidney donation, and (8) increased self-efficacy. Thus, enhancing health literacy in self-management, religious/spiritual approaches, supportive care, and social support played pivotal roles in the recovery and resilience among patients with KT.

## 5. Conclusions

The findings supported five themes related to KTRs’ experiences, which included 25 sub-themes. This study adds a great emphasis on (1) having new life and life satisfaction, (2) fear of kidney rejection and complications, (3) feelings of gratitude and spiritual practices, (4) concerns for high cost to healthcare expenses, and (5) patience with self-management and resilience. Thus, interventions and guidelines for self-management, regarding diet, infection prevention, self-monitoring, social support, and strict follow-up are needed. The supporting physical, psychological, spiritual, and financial dimensions should be taken into consideration. Spiritual and religious beliefs should be applied and implemented in culturally congruent care in healthcare services and also provide information to pre- and post-KT. Additionally, quantitative research and effective interventions are needed for KTRs.

### 5.1. Relevance to Clinical Practice

This study places signifucant emphasis on KTRs having healthier outcomes, returning to everyday life, and achieving life satisfaction, while also addressing the negative impacts of infection, comorbidities, and complications. Interventions and guidelines for self-management, including diet, infection prevention, self-monitoring, and strict follow-up, are necessary. The supporting physical, psychological, spiritual, and financial dimensions should be taken into consideration. Spiritual and religious beliefs should be applied and implemented in culturally congruent care within healthcare services and also provide information to individuals pre- and post-KT. Additionally, nurses can play a vital role in coordinating with the multidisciplinary team to create a case-based program tailored to the KTRs’ needs.

### 5.2. Limitations

The participants in this study were 25 KTRs from the Bhumirajanagarindra Kidney Institute Hospital in Bangkok, Thailand. It could not be generalized to a broader scale in Thailand. Therefore, further studies should be conducted in other regions of Thailand. Additionally, quantitative research and effective interventions are needed for the KTRs.

## Figures and Tables

**Table 1:** Participants’ demographic characteristics (n = 25).

Items	Participant with DDKT	Participant with LRKT
Sex:		
Male	12	1
Female	8	4
Age (year):		
20–30	1	-
31–40	1	1
41–50	2	1
51–60	8	2
61–70	6	1
>70	2	-
Employment status:		
Employed	14	4
Unemployed	2	-
Retirement	4	1
Educational level:		
Elementary	4	1
High school/Diploma	1	-
Baccalaureate	12	4
Masters	2	-
Doctorate	1	-
Cause of CKD:		
HT, DM, DLP	11	1
Autoimmune disease	3	3
Overweight	3	-
Kidney disease (RC, FSGS, PKD)	4	1
Unknown	2	-
Since of KT (ms, ys):		
7 ms–1 y	8	-
>1–2 ys	2	-
>2–3 ys	3	1
>4–5 ys	4	1
>5 ys	4	2
Post KT health condition:		
Infection (CMV, BKV, UTI)	6	1
Complication (Active AMR, DC, LC, RVT, US)	10	-
None	4	4

Note: AMR = Antibody-mediated rejection, BKV = Human Polyomavirus, DC = Disseminated cryptococcosis, DLP = Dyslipidemia, DM = Diabetes mellitus, DDKT = Deceased donor kidney transplant, FSGS = Focal segmental glomerulosclerosis, HT = Hypertension, LC = lymphocele, RVT = Renal vein thrombosis, US = Urethral stricture, UTI = Urinary tract infection.

**Table 2:** The analysis of data revealing five themes, with a total of twenty-five subthemes.

Themes	Subthemes
Having a new life and life satisfaction	1.1 Return to normal life; 1.2 Improving quality of life; 1.3 Having freedom and high energy; 1.4 The best award; 1.5 Returning to work; 1.6 Improved sex life.
2. Fear of kidney rejection and complications	2.1 Worry about kidney rejection; 2.2 Stressed about infection; 2.3 Stressed about complications.
3. Gratitude and spiritual practices	3.1 Appreciation to the donor; 3.2 Appreciation to doctors and nurses; 3.3 Appreciation to family for their support; 3.4 Sense of strangeness, night dream; 3.5 Delusion in the ICU; 3.6 Worship, pray, and do good deeds as in Buddhism; 3.7 To repay the kindness of Donors and those who support; 3.8 Making merit regularly; 3.9 Mindfulness, transcendence meditation, positive thinking.
4. Concerns about the high cost of healthcare expenses	4.1 Cost for KT and readmission; 4.2 Cost of each follow-up; 4.3 Worries about not having enough money.
5. Patience with self-management and resilience	5.1 Monitoring oneself closely; 5.2 Taking a medical regimen, following the services strictly; 5.3 Supportive care; 5.4 Changing daily life activities.

## Data Availability

Data supporting the findings of this study can be obtained from the corresponding author upon reasonable request. Public access to the datasets is restricted to protect the privacy of adolescent participants and to comply with ethical regulations.

## References

[1] The Thai Organ Transplantation Society Report Information on Organ Transplantation for the Year 2023. https://drive.google.com/file/d/1BV05qYumnc7fHaHXv3-cQ9juL9ZCySZm/view.

[2] Antoun J., Brown D.J., Clarkson B.G., Shepherd A.I., Sangala N.C., Lewis R.J., McNarry M.A., Mackintosh K.A., Corbett J., Saynor Z.L. (2023). Experiences of adults living with a kidney transplant-Effects on physical activity, physical function, and quality of life: A descriptive phenomenological study. J. Ren. Care.

[3] Ghelichi-Ghojogh M., Mohammadizadeh F., Jafari F., Vali M., Jahanian S., Mohammadi M., Jafari A., Khezri R., Nikbakht H.-A., Daliri M. (2022). The global survival rate of graft and patient in kidney transplantation of children: A systematic review and meta-analysis. BMC Pediatr..

[4] Wang Y., Hemmelder M.H., Bos W.J.W., Snoep J.D., de Vries A.P.J., Dekker F.W., Meuleman Y. (2021). Mapping health-related quality of life after kidney transplantation by group comparisons: A systematic review. Nephrol. Dial. Transplant..

[5] Ryu J.H., Koo T.Y., Ro H., Cho J.H., Kim M.G., Huh K.H., Park J.B., Lee S., Han S., Kim J. (2021). Better health-related quality of life in kidney transplant patients compared to chronic kidney disease patients with similar renal function. PLoS ONE.

[6] Park S., Park G.C., Park J., Kim J.E., Yu M.Y., Kim K., Park M., Kim Y.C., Kim D.K., Joo K.W. (2021). Disparity in accessibility to and prognosis of kidney transplantation according to economic inequality in South Korea: A widening gap after expansion of Insurance coverage. Transplantation.

[7] Knobbe T.J., Kremer D., Bültmann U., Annema C., Navis G., Berger S.P., Bakker S.J.L., Meuleman Y. (2025). Insights into health-related quality of life of kidney transplant recipients: A narrative review of associated factors. Kidney Med..

[8] De Pasquale C., Pistorio M.L., Veroux M., Indelicato L., Biffa G., Bennardi N., Zoncheddu P., Martinelli V., Giaquinta A., Veroux P. (2020). Psychological and psychopathological aspects of kidney transplantation: A systematic review. Front. Psychiatry.

[9] Ishikawa S., Tasaki M., Saito K., Nakagawa Y., Ikeda M., Takahashi K., Tomita Y. (2023). Long-term CMV Monitoring and chronic rejection in renal transplant recipients. Front. Cell. Infect. Microbiol..

[10] de Boer S.E., Knobbe T.J., Kremer D., van Munster B.C., Nieuwenhuijs-Moeke G.J., Pol R.A., Bakker S.J.L., Berger S.P., Sanders J.S.F. (2024). Kidney transplantation improves health-related quality of life in older recipients. Transpl. Int..

[11] Tucker E.L., Smith A.R., Daskin M.S., Schapiro H., Cottrell S.M., Gendron E.S., Hill-Callahan P., Leichtman A.B., Merion R.M., Gill S.J. (2019). Life and expectations post-Kidney transplant: A qualitative analysis of patients’ responses. BMC Nephrol..

[12] Wube T.B., Asgedom S.G., Mengesha A.G., Bekele Y.A., Gebrekirstos L.G. (2025). Behind the healing: Exploring the psychological battles of kidney transplant patients: A qualitative insight. Health Sci. Rep..

[13] Zerbinati L., Guerzoni F., Napoli N., Preti A., Esposito P., Caruso R., Bulighin F., Storari A., Grassi L., Battaglia Y. (2023). Psychosocial determinants of healthcare use costs in kidney transplant recipients. Front. Public Health.

[14] Birkefeld K., Bauer-Hohmann M., Klewitz F., Kyaw Tha Tun E.M., Tegtbur U., Pape L., Schiffer L., Schiffer M., de Zwaan M., Nöhre M. (2022). Prevalence of mental disorders in a German kidney transplant population: Results of a KTx360°-Substudy. J. Clin. Psychol. Med. Settings.

[15] American Psychological Association Resilience. https://dictionary.apa.org/resilience.

[16] Chung M.H. (2025). Qualitative content analysis of the resilience scale for patients with kidney transplantation. J. Ren. Care.

[17] Creswell J.W., Creswell J.D. (2023). Research Design: Qualitative, Quantitative, and Mixed Methods Approaches.

[18] Heotis E. (2020). Phenomenological research methods: Extensions of Husserl and Heidegger. Int. J. Sch. Cogn. Psychol..

[19] Norlyk A., Martinsen B., Dreyer P., Haahr A. (2023). Why phenomenology came into nursing: The legitimacy and significance of phenomenology in theory building in the discipline of nursing. Int. J. Qual. Methods.

[20] Naeem M., Ozuem W., Howell K., Ranfagni S. (2023). A step-by-step process of thematic analysis to develop a conceptual model in qualitative research. Int. J. Qual. Methods.

[21] Braun V., Clarke V. (2006). Using Thematic Analysis in Psychology. Qual. Res. Psychol..

[22] Wikipedia Gratitude. https://en.wikipedia.org/wiki/Gratitude#cite_note-ReferenceB-4.

[23] Vredeveld P. Gratitude in Buddhism: The Heart of Thanksgiving. https://www.originalbuddhas.com/blog/gratitude-in-buddhism-the-heart-of-thanksgiving?srsltid=ABOor6QvHM_Pzdcl5_ug_3m5fDLsVEYw1KPz_hH0Tz31eXaOVKFFnQ.

[24] SHUKRANA The Common Thread of Gratitude in All Religions. https://www.shukrana.com/gratitude-in-all-religions.

[25] Diniz G., Korkes L., Tristão L.S., Pelegrini R., Bellodi P.L., Bernardo W.M. (2023). The effects of gratitude interventions: A systematic review and meta-analysis. Einstein.

[26] Jiwattanasuk N. (2024). Gratitude to the Buddha: The case study of Buddhamahametta Foundation. J. Sirindhornparidhat.

[27] Abuzar M. (2025). Muslim and Buddhist gratitude practice: A systematic review of psychological benefit for culturally sensitive mental health interventions. Hamdard Islam..

[28] ThaiPBS Understanding the 4 Thai Health Insurance Systems: What Will They Cover in 2025?. https://www.thaipbs.or.th/news/content/352794.

[29] Lorenz E.C., Egginton J.S., Stegall M.D., Cheville A.L., Heilman R.L., Nair S.S., Mai M.L., Eton D.T. (2019). Patient experience after kidney transplant: A conceptual framework of treatment burden. J. Patient-Rep. Outcomes.

[30] Helanterä I., Isola T., Lehtonen T.K., Åberg F., Lempinen M., Isoniemi H. (2019). Association of clinical factors with the costs of kidney transplantation in the current era. Ann. Transplant..

[31] Hangto P., Srisuk O., Chunpeak K., Hutchinson A., van Gulik N. (2022). Effectiveness of a multidisciplinary self-management education programme for kidney transplant recipients in Thailand. J. Kidney Care.

[32] Huang Y., Li S., Lu X., Chen W., Zhang Y. (2024). The effect of self-management on patients with chronic diseases: A systematic review and Meta-analysis. Healthcare.

[33] Lee H., Kang C.M. (2025). Self-management interventions for kidney transplant recipients: A systematic review. Healthcare.

[34] Aderinto N., Olatunji G., Kokori E., Ogieuhi I.J., Moradeyo A., Woldehana N.A., Lawal Z.D., Adetunji B., Assi G., Nazar M.W. (2025). A narrative review on the psychosocial domains of the impact of organ transplantation. Discov. Ment. Health.

[35] Xu J., Zhang L., Sun H., Gao Z., Wang M., Hu M., Ji Q., Guo L. (2023). Psychological resilience and quality of life among middle-aged and older adults hospitalized with chronic diseases: Multiple mediating effects through sleep quality and depression. BMC Geriatr..

[36] González-Flores C.J., García-García G., Lerma A., Pérez-Grovas H., Meda-Lara R.M., Guzmán-Saldaña R.M.E., Lerma C. (2021). Resilience: A protective factor from depression and anxiety in Mexican dialysis patients. Int. J. Environ. Res. Public Health.

[37] Matsumura N., Mizukawa M.K., Hashino A., Kazawa K., Naka M., Huq K.A.T.M.E., Moriyama M. (2024). Factors influencing self-management behaviors among patients with post-kidney transplantation: A qualitative study of the chronic phase transition. Healthcare.

